# Microcrystalline Tyrosine (MCT^®^): A Depot Adjuvant in Licensed Allergy Immunotherapy Offers New Opportunities in Malaria

**DOI:** 10.3390/vaccines5040032

**Published:** 2017-09-27

**Authors:** Gustavo Cabral-Miranda, Matthew D. Heath, Ariane C. Gomes, Mona O. Mohsen, Eduardo Montoya-Diaz, Ahmed M. Salman, Erwan Atcheson, Murray A. Skinner, Matthias F. Kramer, Arturo Reyes-Sandoval, Martin F. Bachmann

**Affiliations:** 1 Nuffield Department of Medicine, Centre for Cellular and Molecular Physiology (CCMP), The Jenner Institute, University of Oxford, Oxford OX3 7BN, UK; ariane.cruzgomes@kellogg.ox.ac.uk (A.C.G.); mona.mohsen@kellogg.ox.ac.uk (M.O.M.); genlalo@yahoo.com.mx (E.M.-D.); ahmed.salman@ndm.ox.ac.uk (A.M.S.); erwan.atcheson@seh.ox.ac.uk (E.A.); arturo.reyes@ndm.ox.ac.uk (A.R.-S.); 2 Allergy Therapeutics (UK) Ltd. Dominion Way, Worthing BN14 8SA, UK; Matthew.Heath@allergytherapeutics.com (M.D.H.); Murray.Skinner@allergytherapeutics.com(M.A.S.); 3 Bencard Allergie GmbH, Leopoldstr, 175, 80804 München, Germany; KramerM@bencard.com; 4 Immunology, RIA, Inselspital, University of Bern, 3010 Bern, Switzerland

**Keywords:** vaccine, malaria, adjuvant, MCT

## Abstract

Microcrystalline Tyrosine (MCT^®^) is a widely used proprietary depot excipient in specific immunotherapy for allergy. In the current study we assessed the potential of MCT to serve as an adjuvant in the development of a vaccine against malaria. To this end, we formulated the circumsporozoite protein (CSP) of *P. vivax* in MCT and compared the induced immune responses to CSP formulated in PBS or Alum. Both MCT and Alum strongly increased immunogenicity of CSP compared to PBS in both C57BL/6 and BALB/c mice. Challenge studies in mice using a chimeric *P. bergei* expressing CSP of *P. vivax* demonstrated clinically improved symptoms of malaria with CSP formulated in both MCT and Alum; protection was, however, more pronounced if CSP was formulated in MCT. Hence, MCT may be an attractive biodegradable adjuvant useful for the development of novel prophylactic vaccines.

## 1. Introduction

Aluminum hydroxide (Alum) is the most widely used adjuvant in vaccinology, in particular for prophylactic vaccines [1,2]. Despite its widespread use, Alum has several drawbacks such as persistence at injection site due to the non-biodegradable nature [3,4,5]. An additional disadvantage of Alum is the stimulation of so-called T-helper type 2 (Th2) as opposed to Th1 immune responses, leading to induction of poorly protective IgG subclasses [6]. There is therefore a need for the development of novel safe and efficacious adjuvants for future vaccines.

Microcrystalline Tyrosine (MCT) was originally developed for use in allergen-specific immunotherapy (SIT) for the treatment of allergy. MCT has been used as a depot for a number of years with indications of its safe use and ability to enhance immune responses [7,8,9]. The proprietary platform exhibits distinct physicochemical properties, distinct mode of adsorption with antigens and an established stability profile, generates robust and sustained IgG antibodies titers with no unusual propensity to stimulate IgE. Its biodegradable nature results in total clearance at injection site (mitigating risk of granuloma formations)—while delivering a sustained release of antigens for prolonged immune exposure and, unlike alum, it is fully metabolized within the body [8,9]. The adjuvant mechanism of MCT is subject to ongoing studies, and has been reported to induce significant Th1 cytokine profiles and activation of specific signaling pathways [9]. Moreover, it offers a compatible platform for the adsorption of a wide range of antigens and other immunomodulators to function as part of an adjuvant systems approach [7]. MCT is used in combination with Monophosphoryl Lipid A (MPL^®^) exclusively in allergy immunotherapy that offers shorter course therapy compared to conventional approaches, the observed immunological synergy and physicochemical compatibility in combining MCT with this Th1 adjuvant has been previously reported [9,10]. Since MCT exhibits the advantage of biodegradability over Alum, we wanted to explore its potential in non-allergy indications, and its applicability in infectious diseases, such as malaria as a suitable alternative biodegradable platform that could be translated across a broader vaccine scope.

Despite a continuous and enormous effort to control malaria, the number of malaria episodes worldwide remains alarming [11,12,13,14]. The most recent WHO estimates (released in December 2015) indicate the occurrence of 214 million cases of malaria in 2015, leading to 438,000 deaths worldwide [14].

The intensity of malaria transmission is related to several factors, such as parasite (species), vector (species, lifespan and preferred target for biting), and the environment (climate, related to the number and survival of mosquitoes) [15,16]. The immune response of the human host is also an important factor for a successful transmission. In general, partial immunity may arise over time upon multiple infections, reducing the risk of having a severe malaria infection but not ensuring full immune protection [17]. Hence, strong efforts are being made for the production of effective prophylactic vaccines [18,19,20,21].

The malaria parasite life cycle in humans begins with the bite of the female Anopheles mosquito, inoculating infective sporozoites into the blood stream [22]. The parasite quickly migrates to the liver, where hepatocytes are infected. Sporozoites express the circumsporozoite protein (CSP) on their surface, which bind to receptors on hepatocytes, allowing cellular infection. CSP is therefore the most pursued target for antibody-based vaccines against the parasite’s liver stage. The regionally approved and most advanced vaccine candidate against malaria is the vaccine RTS,S/AS01 which is in clinical development at Phase 3, based on the *P. falciparum* CSP. A large number of studies have shown that the immunization with RTS,S vaccine induces strong and protective antibodies anti-CSP and CD4^+^ T-cells responses. However, RTS,S does not induce significant CD8^+^ T-cell immune responses [23,24,25] 

In the current study, we assessed the ability of MCT to enhance protective immune responses against a CSP based *P. vivax* vaccine. MCT enhanced specific IgG responses and protection in a recombinant *P. vivax/P. bergei* model was more pronounced than protection induced by CSP formulated in Alum, correlating with the induction of more protective IgG subclasses.

## 2. Material and Methods

### 2.1. Procedures Involving Animals

All animals and procedures were used in accordance with the terms of the United Kingdom Home Office and under regulation of The Animals (Scientific Procedure) Act 1986. The Project License was approved by the University of Oxford Animal Care and Ethical Review Committee (PPL 30/2947). The mice were housed in ventilated cages, under specific pathogen free conditions, constant temperature, humidity and with a 12:12 light-dark cycle. For induction of short-term anesthesia, mice were anesthetized using vaporized IsoFlo. All animals were humanely sacrificed at the end of each experiment by an approved Schedule 1 method (cervical dislocation).

### 2.2. Construction and Expression of rPvCSP-c

The construction of the chimeric (rPvCSP-c) protein comprised the clone DNA sequence in open reading frame of the central repeats from VK247 (GenBank P08677) and VK210 (GenBank M69059.1) isoforms, between N and C terminal regions from Salvador 1 strain of *P. vivax* (GenBank 5472322) [26] into pHLsec mammalian expression vector [27]. The chimeric vivax sequence was under the expression control of chicken β-actin/rabbit β-globin hybrid promoter. The pHLsec vector contains a signal sequence for secretion of the protein fused with a C-terminal 6× His-tag. The Endotoxin-Free Plasmid Giga Kit (Qiagen, Manchester, UK) was used to purify recombinant plasmid DNA from E. coli DH5α strain. The recombinant plasmid (pHLsec + VK247-VK210) was transfected into HEK 293T (ATCC CRL-11268) cells to express the chimeric protein PvCSP 210-247. For that, HEK 293T cells were cultured in Roller Bottles (2125 cm^2^) for 72 h (on reaching 90% confluence) at 37 °C, using Dulbecco’s Modified Eagle’s Medium (DMEM High glucose, Sigma, Gillingham, UK) supplemented with L-glutamine, non-essential amino-acids (Invitrogen, Leicestershire, UK) and 10% Fetal Bovine Serum (FBS, Invitrogen). After that, half a milligram of purified plasmid DNA was transfected using 3.6 mL of polyethylenimine (1 mg·mL^−1^) (DNA:PEI complexes) in serum-free DMEM. The HEK 293T cells transfected were cultured for 7 days to express/secret the *P. vivax* CSP protein and the supernatant was used to purify protein.

### 2.3. Purification of rPvCSP-c

The CSP 210-247 chimeric protein was purified from HEK 293T cells’ supernatant with Ni Sepharose excel using an immobilized metal ion affinity chromatography (IMAC) medium (resin) pre-charged with nickel ions as described earlier [26,28]. The conditioned media was filtered through a 0.45 μm membrane (Merck Millipore, Darmstadt, Germany). IMAC purification was performed using a wash step with 5 column volumes (CV) of distilled water (flow velocity: 100 cm/h), equilibration step with 5 CV of equilibration buffer (20 mM sodium phosphate, 0.5 M NaCl, pH 7.4/flow velocity: 150 cm/h), load sample step (flow velocity: 150 cm/h), wash step with 20 CV of wash buffer (20 mM sodium phosphate, 0.5 M NaCl, 20 mM imidazole, pH 7.4/flow velocity: 150 cm/h), linear elution step with 2 CV of 7% elution buffer (20 mM sodium phosphate, 0.5 M NaCl, 500 mM imidazole, pH 7.4/flow velocity: 150 cm/h) and 2 CV of 70% elution buffer (flow velocity: 150 cm/h). Elution samples after IMAC purification were submitted to 12.5% SDS-PAGE under reducing conditions and proteins were visualized with Silver stain and Western blot analyses using the monoclonal antibody (MRA-1028K sporozoite ELISA kit, Thermo Fisher Scientific, Loughborough, UK) anti-VK 210 and -VK 247. The positive samples were selected to concentrate using Amicon^®^ ultra centrifugal filters system (Life technologies, Warrington, UK) until 10 mL of final volume. The concentration of the recovery sample was tested with Bradford protein assay and the purity with Silver and Coomassie staining. Contaminating proteins were removed with size exclusion purification (SEC). For that, the Superdex 200 SEC medium (GE) was used in the column. We used 10 CV of PBS (10 mM phosphate buffer, 140 mM sodium chloride, pH 7.4) in the equilibration step. The sample was eluted isocratically from a SEC column, using a PBS buffer and the flow rate in the both steps was 0.5 mL/min. The ÄKTA purifier was using for IMAC and SEC process.

### 2.4. Vaccine Formulation Using MCT and Aluminum Hydroxide Adjuvant (Alum) as Adjuvant Plus rPvCSP-c Protein

The MCT vaccine preparation was based on the Allergy Therapeutics (Worthing, UK) Ltd experience for allergen-formulation. For that, a target concentration of 2% (20 mg/mL) of MCT was attained through diluted with PBS and mixed to the antigen (rPvCSP-c) in PBS buffer. The injection was administered intramuscularly immediately after the vaccine formulation.

For the Alum vaccine preparation, it was according to the manufacturer’s specifications (InvivoGen, San Diego, CA, USA). Briefly, the Alum (10 µg of Al^3+^ per dose) was mixed with 20 mM TRIS buffer (pH 7.0–7.5) and left at room temperature (RT) for 15 min, subsequently added rPvCSP-c protein to the corresponding eppendorf tubes containing Alum + TRIS buffer and vortexed gently for five seconds. After that, the vaccine was incubated at RT for one hour and vaccinated intramuscularly as soon as possible within the same day.

To assess the protein adsorption to adjuvant, the vaccine (either CSP/2%MCT and CSP/Alhydrogel) was centrifuged at 3000 rpm for 10 min and protein content of non-adsorbed adjuvant in supernatant was determined using Bradford method with standard protein concentration range 10 µg/mL–500 µg/mL using spectrophotometer at wavelength ratio of absorbance 590 nm/470 nm. Amount of CSP adsorbed on the adjuvants was determined by deducting amount of CSP detected in supernatant from total protein (CSP at 150 µg/mL in Tris buffer).

### 2.5. First Experimental Design to Test the Immunogenic Capacity of MCT for Malaria Vaccine Development Using rPvCSP-c as Antigen

In order to assess the capacity of MCT humoral immunity in vaccine development for *P. vivax* malaria, a schedule of vaccination was designed that consisted 2 vaccinations with 2 different doses of proteins chimeric CSP 210-247 (5 µg each dose) formulated in MCT at 2% (20 mg/mL), prime (day 0) and boost (day 14). As control, a group of mice was immunized with CSP 210-247 protein diluted in sterile PBS without adjuvant and another group with PBS. Six mice per group age-matched 6 weeks-old female inbred C57BL/6 strain were vaccinated intramuscularly with 50 μL dose of each vaccine.

### 2.6. Assessment of Antibody Production (IgG total) of C57BL/6 Mice Vaccinated with rPvCSP-c with or without Adjuvant (MCT)

The humoral immune response was analyzed by ELISA (Enzyme-Linked Immunosorbent Assay). A 96-well micro titer ELISA plates (Thermo Fisher Scientific, Loughborough, UK) were coated with 100 µL per well at 1µg/mL of CSP 210-247 diluted in carbonate buffer (CBB) 50 mM at pH = 9.6 and incubated overnight at 4 °C. To avoid non-specific binding, the wells were filled with 200 μL of blocking solution (5% Skim Milk Powder (Sigma-Aldrich, Gillingham, UK) in PBS with 0.05% tween 20) at RT for 2 h, then flick-washed 3× with PBS 0.05% tween. The samples (sera) were then add (50 µL per well) in triplicate, diluted 1:100 in PBS 0.5% skimmed milk, then incubated at RT for 2 h, washed 3× with PBS 0.05% tween, and 50 μL applied per well of detecting antibody (Ab), goat anti-mouse IgG diluted 1:2000 (Secondary Antibody, HRP conjugate (ThermoFisher, Loughborough, UK)), and incubated for 1 h at RT. For detection of IgG subclasses, subclass specific secondary antibodies were used diluted 1:1000 (Goat anti-mouse IgG1, IgG2a, IgG2b, IgG3, HRP coupled, Life Technologies). Then, 70 μL/well of TMB substrate (Sigma-Aldrich) was added and incubated at RT for 15 min, the reaction was stopped with 0.5 M H_2_SO_4_ (70 μL/well) and the plate read using microplate reader at 450 nm.

### 2.7. Vaccination Schedules Using rPvCSP-c Conjugated with MCT and Comparing it with Aluminum Hydroxide

After testing the immunogenic capacity of MCT as adjuvant for vaccine *P. vivax* malaria development, an experiment was subsequently designed (Figure 3) using BALB/c mice instead of C57BL/6 strain, in order to assess the immunological capability of MCT to different species of mice. Besides that, as we intended to challenge the mice with parasite, a higher concentration of protein (7.5 µg) formulated using 3 vaccinations with MCT at 2% (20 mg/mL), prime (day 0), first boost (day 14) and second boost (day 28). In this new experiment, Alum was included in parallel, as previously described. As negative control, mice were immunized with CSP 210-247 protein diluted in sterile PBS without adjuvant and in the other group only PBS. Six mice per group age-matched 6-weeks-old female inbred BALB/c were vaccinated intramuscularly with 50 μL dose of each vaccine.

### 2.8. Isolation of Parasites and Challenge

*P. bergei* replacement expressing *P. vivax* CSP 210-247 was used to challenge the mice. As previously detailed [26], this parasite was previously tested and exhibited high efficacy to infect Balb/c mice, which was confirmed in this study. Moreover, the parasite is constructed in a very similar way to the chimeric parasite *P. bergei* replacement expressing *P. vivax* TRAP [29]. At the insectary of the Jenner Institute, female Anopheles stephensi mosquitoes were fed on infected Tuck-ordinary (TO) inbred mice. Briefly, exflagellation was first confirmed, and mosquitoes were exposed to anesthetized infected mice for 15 min. The mosquitoes were then maintained for 21 days in a humidified incubator at a temperature of 19 to 21 °C on a 12-h day-night cycle and fed with a fructose-p-aminobenzoic acid (PABA) solution. At 21 days, salivary glands were dissected from mosquitoes into Schneider’s media (Pan Biotech, Aidenbach, Germany) and sporozoites gently liberated using a glass homogenizer. Sporozoites were then diluted to the required concentration for 1000 parasites in 100 µL and intravenously injected into the tail vein of the mouse. Two independent challenge experiments with six female BALB/c mice per arm were performed.

### 2.9. Statistical Modeling to Predict Parasitaemia

Percent parasitaemia was used to calculate the time required to reach a blood-stage infection of 1% or time to 1% parasitaemia. This was predicted using a linear regression model as described previously [26,28,30,31]. Briefly, blood parasite counts were obtained for 6 consecutive days starting on day 4 after the challenge. Blood smears were stained with Giemsa and percentages of parasitaemia calculated in all animals. The logarithm to base 10 of the calculated percentage of parasitaemia was plotted against the time after challenge and Prism 5 for Mac OS X (GraphPad Software, La Jolla, CA, USA) statistical analysis package used for generating a linear regression model on the linear part of the blood-stage growth curve.

### 2.10. Statistical Analysis

For all statistical analyses, GraphPad Prism version 5.0 for Max OS was used unless indicated otherwise. Prior to statistical analysis to compare two or more populations, the Kolmogorov–Smirnov test for normality was used to determine whether the values followed a Gaussian distribution. An unpaired *t*-test was employed to compare two normally distributed groups, whereas Mann–Whitney rank test was used for comparing two non-parametric groups. If more than two groups were present non-parametric data was compared using Kruskal–Wallis test with Dunn’s multiple comparison post-test, whereas normally distributed data were analyzed by one-way ANOVA with Bonferroni’s multiple comparison post-test. The effect of two variables was explored using two-way ANOVA with Bonferroni’s multiple comparison post-test. Correlation strength was tested using either Pearson’s or Spearman’s tests as indicated in the results chapters. Kaplan–Meier survival curves were used to represent protective efficacy to a challenge with any *P. berghei* parasite lines. All ELISA titers were also log10 transformed before analysis. The value of *p* < 0.05 was considered statistically significant (* *p* < 0.05, ** *p* < 0.01, ** *p* < 0.001 and *** *p* < 0.001).

## 3. Results and Discussion

### 3.1. Production and Characterization of Purified Recombinant rPvCSP-c and Vaccine Formulation

**Figure 1:**
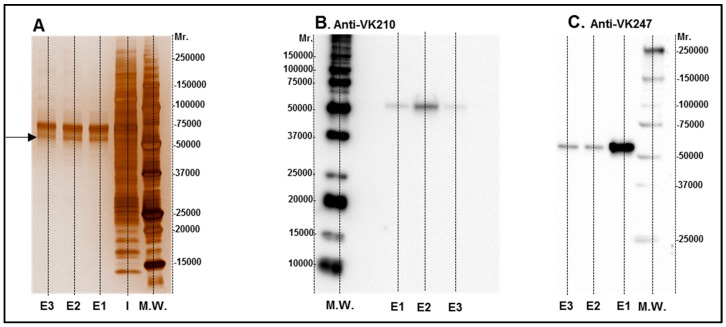
Images of rPvCSPc in silver staining (**A**) and Western blot (**B**,**C**). Elution samples after IMAC purification were submitted to 12.5% SDS-PAGE under reducing conditions and proteins were visualized with Silver stain (**A**). Lane I: Input of sample. E1 to E3 elution fractions were selected for SEC purification and tested with Western blot analyses using monoclonal antibodies for VK 210 (**B**) and VK 247 (**C**).

HEK 293 T cells were transfected with a plasmid encoding HIS-tagged CSP 210-247 protein, which is consisting of the central repeats the VK247 and VK210 variats. The chimeric protein was purified from HEK 293T cell supernatant with a Ni-column and samples were analyzed by 12.5% SDS-PAGE under reducing conditions and proteins were visualized using Silver stain (Figure 1A). Protein identity in purified samples was confirmed using CSP210- and CSP247-specific monoclonal antibodies (Figure 1B,C). As expected, the chimeric CSP protein had a molecular weight of about 55 kDa as estimated by comparison with known molecular weight standards (M.W.). After protein identification (Figure 1), the contaminating proteins were removed by SEC, and the purified protein was concentrated to be used for vaccine formulation and immunoassays. The CSP is an antigen considered to be extremely abundant on the parasite surface which allows the cellular infection by sporozoites. Moreover, CSP is highly immunogenic and one of the most studied epitope of *Plasmodium*. Particularly in *P. vivax* the VK247 and VK210 variants are present woodwind and are highly conserved [32] which make the use of a chimeric protein joining these two variations extremely attractive for vaccine development.

### 3.2. Humoral Immune Response in C57BL/6 Mice Induced by rPvCSP-c Formulated in MCT

**Figure 2:**
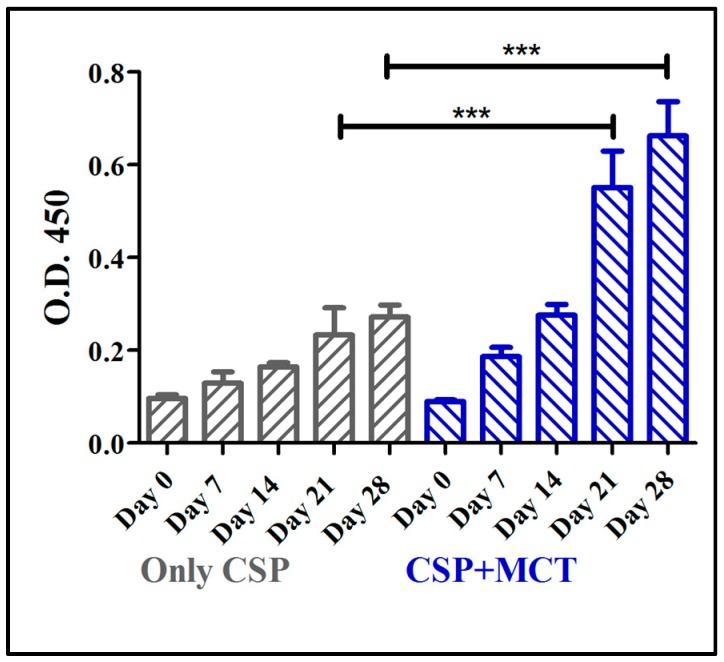
Mouse whole IgG ELISA against chimeric CSP 210-247. C57BL/6 mice were immunized with 5 µg CSP + 2% MCT or only CSP two times, prime (day 0) and boost (day 14); and blood was taken weekly from day 0 (before the prime) until day 28, two weeks after the boost. Antibodies production (whole IgG) increased from day 7 to day 28 in both group. Titers were statistically significantly different between the groups on days 21 and 28.

The CSP antigen was formulated with MCT at a concentration of 2% that is routinely used in commercial allergy vaccines. Groups of C57/BL/6 mice were immunized with CSP either formulated in MCT or PBS and specific IgG responses were assessed by ELISA. MCT enhanced IgG responses after the first boost (day 21) and responses remained higher for the remainder of the experiment (Figure 2). Differences between antibody levels on day 21 and day 28 were statistically significant (*p* = 0.0006 on day 21 and *p* = 0.0001 on day 28). This result prompted us to study the adjuvant capacity of MCT in more detail and compare it to Alum.

### 3.3. Humoral Immune Response of BALB/c Mice Vaccinated with rPvCSP-c Formulated in MCT, Alum or without Adjuvant

**Figure 3:**
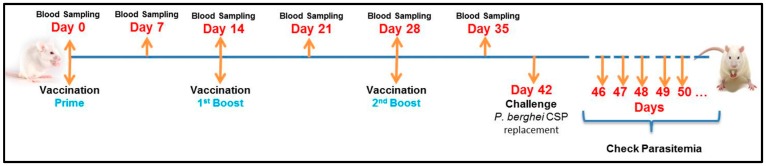
Experimental Design. BALB/c mice were vaccinated intramuscularly with 7.5 µg of CSP for each dose (50 μL). The vaccinations were done 3 times: prime (Day 0), first boost (Day 14) and second boost (Day 28). The samples (sera) were collected weekly, starting before the first vaccination (Day 0) and up to Day 35 (one week before the challenge). On day 42 the mice were challenged with *P. berghei* expressing CSP of *P. vivax*. As described before, the parasitemia was checked daily beginning in the 4th day after challenge until the mice get 1% parasitaemia, when they were euthanized.

In a next set of experiments, we compared the adjuvants properties of MCT with the classical adjuvant Alum. Figure 3 outlines the immunization schedule. To assess whether our findings in C57BL/6 mice hold true for other mouse strains, we used BALB/c mice for immunization.

**Figure 4:**
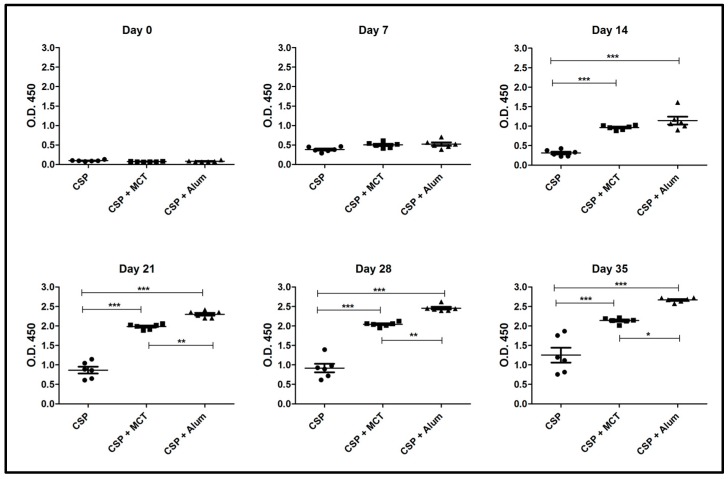
ELISA results of total IgG against CSP 210-247. Groups of mice were immunized as shown in Figure 3. Group 1 was vaccinated with only CSP without adjuvant, group 2 received CSP + MCT adjuvant and group 3 CSP + Alum (aluminum hydroxide). Six BALB/c mice per group were vaccinated intramuscularly with 7.5 μg of each vaccine and bleed weekly. The results were analyzed using GraphPad Prism software applied to assess the means of groups by Tukey’s Multiple Comparison Test of one-way ANOVA. *** *p* value < 0.0001; ** *p* value < 0.001; * *p* value < 0.01.

As seen in the previous experiment, MCT significantly increased the IgG response from day 14 against rPvCSP-c also in BALB/c mice. Both MCT and Alum generated high and sustained IgG antibody titers at all time points when compared to rPvCSP-c formulated in PBS (Figure 4). At later time-points (from Day 21) Alum produced a higher IgG response than MCT. Thus, MCT enhanced IgG responses against the chimeric CSP-protein in a mouse strain-independent fashion

Anti-CSP IgG is the most important goal of a CSP-based vaccines, because CSP-specific antibodies have been associated and used to predict vaccinate efficacy for a long time. Indeed most studies have shown that the main focus of vaccine development using CSP is based on antibody production, for example, the approved vaccine candidate against malaria, RTS,S/AS01 [23,24,25]. The RTS,S vaccines induces robust and protective antibodies against CSP but not CD8^+^ T-cells responses seem to be induced. Moreover, the level of IgG as well as the quality and subclasses of the antibodies are particularly important, because the sporozoites circulate for a short time only after a mosquito bite.

**Figure 5:**
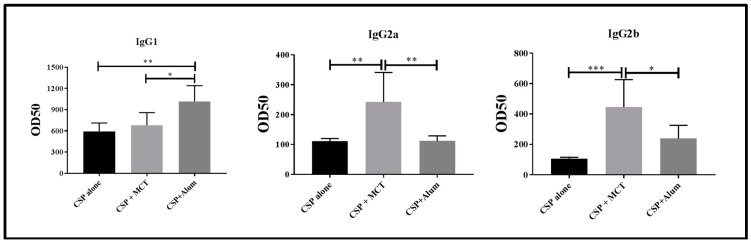
Assessment of relevant subclasses of IgG antibodies (IgG1, IgG2a, IgG2b). Mice were immunized as described in Figure 3 and Figure 4 and subclass specific IgG responses were determined on day 35. The results were analyzed using GraphPad Prism software applied to compare the means of groups by Tukey’s Multiple Comparison Test of one-way ANOVA. *** *p* value < 0.0001; ** *p* value < 0.001; * *p* value < 0.01.

For these reasons, we determined IgG subclasses induced by vaccination one week after the second boost (Figure 5). CSP alone and CSP in Alum induced IgG responses strongly dominated by IgG1. In contrast, MCT-formulated CSP also induced a strong IgG1 response but IgG subclasses were more balanced as IgG2a and IgG2b were markedly higher compared to the other two vaccine formulations. Most protective IgG subclasses against sporozoite infection are IgG2a and IgG2b in mouse [30]. Both these subclasses have been found mostly in the CSP/MCT group. Antibody subclasses induced by vaccination may also be used to identify the type of T helper cells that have been generated; in the case of MCT, it is Th1, which is associated with malaria protection. Indeed, MCT’s ability to induce Th1 cells and Th1 cytokine profiles as well as activation of specific signaling pathways has previously been reported [9,31].

### 3.4. Protective Capacity of Vaccines Subsequent to Challenge with Recombinant P. berghei Expressing CSP of P. vivax

In contrast to *P. berghei*, *P. vivax* does not infect mice and challenge experiments therefore cannot be performed using *P. vivax* itself. To bypass this problem, we used recombinant *P. berghei* sporozoites expressing CSP of *P. vivax* (described in detail elsewhere [26]). Briefly, the endogenous PbCSP gene of the transgenic *P. berghei* was irreversible replaced with its ortholog from *P. vivax*, which possibilities the test of vaccine which target CSP of *P. vivax*. This new transgenic *P. berghei* was fully tested for infection capacity and was found not to diminish the parasite’s ability to replicate within mosquitoes and vertebrate host. The ability to infect the liver was also investigated and it showed a similarly capacity as wild-type sporozoites to cause parasitemia. 

**Figure 6:**
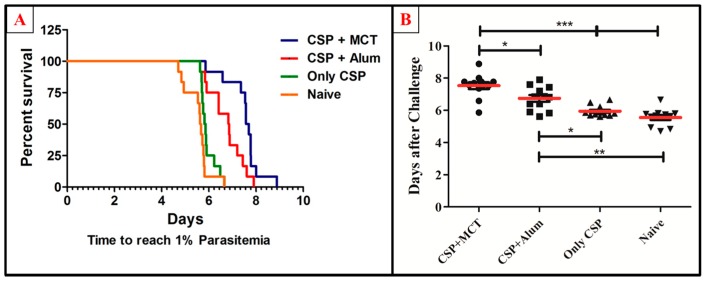
Survival curve and comparative analysis of challenge with *P. berghei* expressing CSP of *P. vivax*. Mice were immunized as described in Figure 3 and challenged on day 42. (**A**) Kaplan Meyer curve for the time until mice have more than 1% of the erythrocytes infected. (**B**) Number of days until mice have more than 1% of erythrocytes infected. MCT + CSP induced significantly better protection than the group vaccinated with CSP formulated in Alum (*p* = 0.0148). Moreover, CSP formulated in MCT induced highly significant protection compared to control mice(*p* = 0.0001). The data represent pooled results of 2 independent experiments (6 mice per group in each experiment), and were analyzed using GraphPad Prism software applied to assess the means of groups by Tukey’s Multiple Comparison Test of one-way ANOVA. *** *p* value < 0.0001; ** *p* value < 0.001; * *p* value < 0.01.

**Figure 7:**
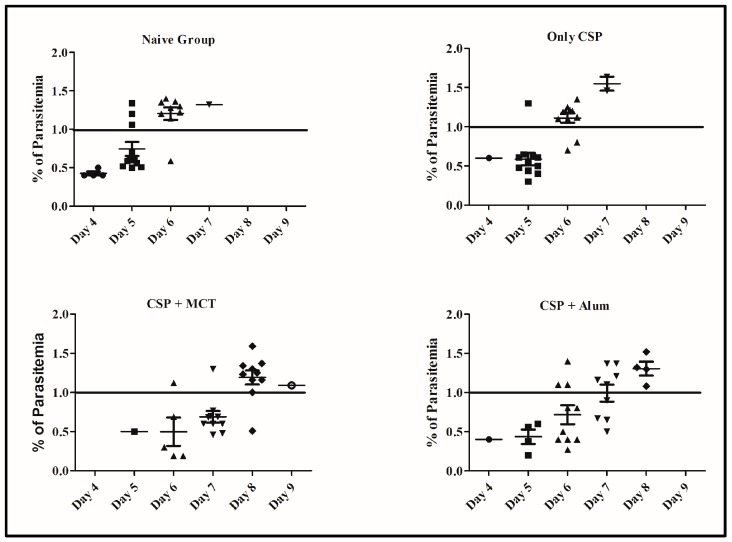
Percentage of erythrocyte infection at each time points for each group. Parasitemia levels were determined at individual time points and shown for each mouse from Figure 6.

To test the potency of the different CSP-formulations, age-matched 6-weeks-old female BALB/c mice were vaccinated (a total of twelve mice per group in 2 independent experiments) and challenged intravenously with 1000 recombinant Plasmodium sporozoites. MCT/rPvCSP-c induced protection that was statistically significantly better than protection by the vaccine formulated in Alum (Figure 6). To better understand the development of parasitemia, we also plotted frequency of infected red blood cells for each time point of each group (Figure 7). The group vaccinated with PBS (Naïve) and the one vaccinated with protein only did not survive more than 7 days after challenge. The Naïve group had only one mouse that survived until day 7, while the CSP group had 2; all other mice reached 1% or more before day 7. Within the groups immunized with protein + adjuvant, only the group vaccinated with CSP + MCT had mice that survived until Day 9 after challenge and protection was significantly better compared to the Alum group (*p* < 0.02).

Several possibilities may exist to explain why MCT groups conferred better protection efficacy. It is known that CSP-specific IgG2a/IgG2b antibodies are more relevant at protecting against challenge infection [33]. As CSP in MCT induced higher levels of specific IgG2a/IgG2b than CSP in Alum, the difference in subclasses induced may be a good candidate for the improved protection induced by CSP in MCT. In addition, the notion that MCT may preserve the native structure of CSP better than Alum, resulting in the induction of more protective antibodies, remains an additional possibility as Alum has been reported to partly denature influenza hemagglutinin [6,34]. The predominant mode of antigen adsorption between MCT and alum is distinct and has been characterized elsewhere [7,9]. We are currently exploring this notion of integrity using a variety of in vivo and in vitro assays.

## 4. Conclusions

MCT is one of the few depot adjuvants formulations used in licensed vaccines for use in humans but not been tested in many infectious disease models. Here we demonstrate that MCT is able to produce robust IgG responses, with significant protective efficacy against the sporozoite of *P. vivax* conferred. These results may suggest that MCT could be used more broadly in prophylactic vaccine development and may be able to serve as an alternative biodegradable platform to the more commonly used Alum.
